# Improved content aware scene retargeting for retinitis pigmentosa patients

**DOI:** 10.1186/1475-925X-9-52

**Published:** 2010-09-16

**Authors:** Walid I Al-Atabany, Tzyy Tong, Patrick A Degenaar

**Affiliations:** 1 Department of Bioengineering, Imperial College London, London, UK; 2 Department of Computing, Imperial College London, London, UK; 3 School of Electrical, Electronic and Computer Engineering, Newcastle University, UK; 4 Department of Biomedical Engineering, Helwan University, Egypt

## Abstract

**Background:**

In this paper we present a novel scene retargeting technique to reduce the visual scene while maintaining the size of the key features. The algorithm is scalable to implementation onto portable devices, and thus, has potential for augmented reality systems to provide visual support for those with tunnel vision. We therefore test the efficacy of our algorithm on shrinking the visual scene into the remaining field of view for those patients.

**Methods:**

Simple spatial compression of visual scenes makes objects appear further away. We have therefore developed an algorithm which removes low importance information, maintaining the size of the significant features. Previous approaches in this field have included *seam carving*, which removes low importance seams from the scene, and *shrinkability *which dynamically shrinks the scene according to a generated importance map. The former method causes significant artifacts and the latter is inefficient. In this work we have developed a new algorithm, combining the best aspects of both these two previous methods. In particular, our approach is to generate a *shrinkability *importance map using as seam based approach. We then use it to dynamically shrink the scene in similar fashion to the *shrinkability *method. Importantly, we have implemented it so that it can be used in real time without prior knowledge of future frames.

**Results:**

We have evaluated and compared our algorithm to the *seam carving *and image *shrinkability *approaches from a content preservation perspective and a compression quality perspective. Also our technique has been evaluated and tested on a trial included 20 participants with simulated tunnel vision. Results show the robustness of our method at reducing scenes up to 50% with minimal distortion. We also demonstrate efficacy in its use for those with simulated tunnel vision of 22 degrees of field of view or less.

**Conclusions:**

Our approach allows us to perform content aware video resizing in real time using only information from previous frames to avoid jitter. Also our method has a great benefit over the ordinary resizing method and even over other image retargeting methods. We show that the benefit derived from this algorithm is significant to patients with fields of view 20° or less.

## Background

There are thought to be 38 million people suffering from blindness worldwide, and this number is expected to double over the next 25 years [1]. Additionally, there are more than 124 million people who have severely impaired vision. The low vision pathologies of this latter group can be divided mainly into two categories; those that predominantly suffer from a loss of visual acuity such as Macular Degeneration (MD), and those that predominantly suffer from a reduction in the overall visual field, such as Retinitis Pigmentosa (RP). RP in particular (population prevalence ~1:4000 [2]) causes a tunnel vision with decreasing peripheral fields as the condition progresses.

For those with central visual impairment, conventional low vision aids (LVAs) can provide magnification in order to compensate for reduced visual acuity. Also, electronically enhanced visual aids have been proposed which offer a number of distinct advantages over conventional LVAs by enhancing the contrast without the need of image magnification [3-6].

Severe visual field (VF) impairment (those with a 20° in remaining tunnel or worse) can greatly affect a patient's mobility and navigation. Despite ongoing research into genetic and pharmacological therapies [7], there is currently no effective treatment for RP patients which can significantly slow or arrest the disease. Traditional low-vision aids for these patients have included de-magnifying optics to expand the remaining visual field of those patients. However, such demagnification comes at the cost of optical (fish-eye) distortion and a loss of resolution (i.e. the objects seem more distant).

Recently, Peli et. al. developed an augmented vision system [8] which multiplexing minified edges over the original scene on a see-through display. However, there is the potential for inattentional blindness, which is the inability of observers to maintain awareness of events in more than one of two superimposed scenes [9].

This paper introduces a new method for image retargeting for those with peripheral vision impairment without degrading the resolution or adding more complexities to the visual scene.

Image resizing is an interesting topic in the image processing field, due to the increasing demand for displaying images and videos on a variety of display devices of different resolutions or aspect ratios. Standard image resizing techniques, such as scaling and cropping, are not efficient. Scaling is applied uniformly by reducing the sampling over the whole image. As with its optical (demagnifying) counterpart, it results in the key features becoming smaller and appearing further away. An alternative approach is to use scene cropping, as performed by Suh et al. [10] and Chen et. al. [11], which involves finding the best rectangular sub-window in the image to be cropped. This is useful only if there is a single important feature in the image then the image can be cropped and scaled to fit. However, Images with multiple important features present a more challenging case for image cropping.

Recently, important progress has been achieved in the development of content-aware image and video resizing techniques. Liu and Gleicher [12] proposed a different image retargeting algorithm, which determines a region of interest (ROI) and then applies a novel fisheye-view warping that applies a piecewise linear scaling function in each dimension to the image to achieve a target image size. Their algorithm is simple, but the warping may cause distortions that look unnatural.

Setlur et al. [13,14] proposed an alternative approach for retargeting large images to small size displays by segmenting the proposed image into a background layer and different ROIs objects, then cuts the ROIs from the image, and fills the holes with an inpainting scheme. Then it rescales the background image and finally pastes the ROIs back to the image. Despite the quality of the resized image, this approach relies strongly on the quality of segmentation which can be difficult, requiring complex feature recognition to be able to be performed properly.

Avidan and Shamir [15] recently provided a new algorithm called *seam carving*. This algorithm alters the dimensions of an image by removing a connected path of pixels, called a seam, from an input image repeatedly to achieve a target size. Although the technique shows good results for static images, it has limitations. When an image is overly compressed, the algorithm starts to carve out important objects, yielding unnatural artefacts.

Extending *seam carving *to video, by treating each frame as an image and resizing it independently, creates jittery artefacts due to the lack of temporal coherency. Rubinstein et al. [16] improved the algorithm by treating video as a 3D cube and extending *seam carving *from 1-D paths to 2-D manifolds in a 3-D volume. There are two limitations; Firstly, if there are moving objects which travel from top to down and from left to right, then it will be so difficult to find a 2-D plane that avoid crossing these objects causing unnatural artefacts. Secondly, this algorithm cannot process real time video, and is therefore not useful for those with restricted field of view.

Wolf et al. [17] proposed a system to retarget video by using non-uniform global warping. Given an input image, their algorithm first computes the importance of each pixel, based on spatial edges, face detection and motion detection. Then, based on the *importance *map, it forms a system of linear equations solved by a least squares manner.

A similar system was later proposed by Zhang et al. [18], where they used the same *importance *map of the Wolf method, but they then used it to calculate a shrinkability matrix. Each pixel of the image is compressed according to it is shrinkage value. This algorithm shows fewer artefacts than the *seam carving *method because it shrinks the image rather than removing pixels. However, the compression efficiency of this method in keeping the original size of the important objects while minimizing less important features is low.

Kim et al. [19] recently provided a strip based image and video retargeting technique. Their approach divides the image into strips and compresses each strip individually based on the gradient complexity within each strip. The advantage with this approach is that it can more uniformly distribute the compression across the image. However, this technique can distort the objects in the image. This is because part of one object may be scaled with a scaling factor different to the other parts when an object resides between two strips. Furthermore, this algorithm is not efficient in video as moving objects can cross different stripe boundaries causing significant artefacts in the resultant target video.

From a comprehensive review of the literature it is clear that there are some trade-offs in these prior methods. In this paper we combine the advantages of the *seam carving *and the pixel *shrinkability *method, by designing a novel *importance *map based on the *seam carving *which we use to shrink the pixels. Also, we show how our method can be scalable to perform content aware video resizing in real time. This is possible as our method only needs to consider previous rather than future frames.

## Methods

**Figure 1:**
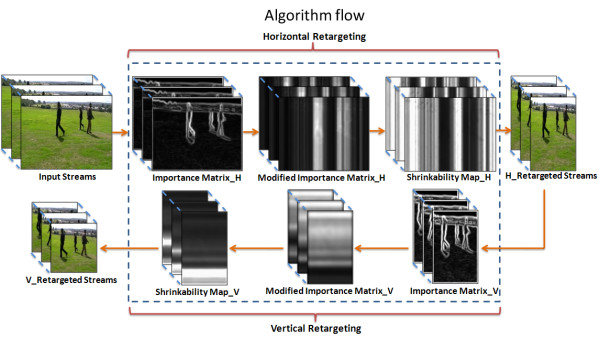
**Algorithm flow**. This diagram illustrates the major steps in our algorithm, the top raw shows the processing in the horizontal direction and the bottom raw shows the vertical one.

Our system, described in Figure 1, consists of five main steps;

1. A per-pixel importance matrix is computed indicating the significance of each pixel. This is a combination of two measures: a local saliency gradient map, and a block difference motion detector map.

2. A modified importance map is computed based on the seam pixel locations.

3. A shrinkage map is computed from the modified importance map.

4. This shrinking map is scaled to resize the whole image to the desired k columns.

5. Finally, a remapping algorithm is applied to re-map each pixel in the original image into its new location in the retargeted image. Thus, a pixel with a low shrinking value is mapped to a distance of approximately one from its left neighbor, while a pixel of high shrinking value is mapped closer to its neighbor.

These five steps are repeated for the vertical resizing.

### A) Generating the importance matrix

Our method of generating an *importance *matrix has similarities to that of Wolf [17]. However, to calculate the saliency map, we use a pyramidal edge detection method to improve computational efficiency. Each pixel in the *importance *matrix is a combination between the saliency of the current pixel in the source image and the dynamic information of this pixel compared to its location in the previous frame. Values range between 0 and 1, where 0 refers to non- important pixels and 1 refers to high importance pixels.

#### 1) Spatial saliency map

To obtain the spatial saliency map, we use an algorithm described previously by Fleck [20] which based on a modified Canny filter [21]. Briefly, simple masks [-1, 0, 1] are used to compute the first derivative in four directions: *H *(horizontal), *V *(vertical), *D1*, and *D2 *(diagonal). The *X *and *Y *spatial gradients are then computed by projecting the diagonal differences on both axes.

(1) $$X=H+\frac{D1+D2}{2}$$

(2) $$Y=V+\frac{D1-D2}{2}$$

The amplitude of the spatial gradient is:

(3) $$Ws=\sqrt{X^{2}+Y^{2}}$$

As simple high frequency (small kernel) derivatives of this form can be lossy in their boundary detection, we therefore use a multi-scale pyramidal approach with three kernel sizes to obtain lower frequency (large kernel) spatial derivatives [22].

#### 2) Temporal saliency map

A Motion saliency map is a map used to identify moving objects. Because the human eye is very sensitive to motion, retargeting dynamic scenes while preserving the temporal context is very important. Motion is detected based on a method proposed by Liu et al. [23], which is relatively easy to implement and requires low computational power. The image is divided into NxN blocks and motion in each block is calculated by taking the weighted average of intensity difference of each pixel, so that the motion map *W_T_*(*x*, *y*) is set to one if the block containing the pixel (*x*, *y*) has motion, and zero otherwise. We uses N = 4 in all the processed images and videos in this paper.

The combined spatio-temporal *importance *matrix then is the combination of the spatial and temporal saliency maps, normalised to between 0 and 1.

(4) $$W_{ST}=W_{S}+W_{T}$$

(5) $$W_{ST}=\frac{W_{ST}-\min(W_{ST})}{\max(W_{ST})-\min(W_{ST})}$$

### B) Modifying the importance matrix

**Figure 2:**
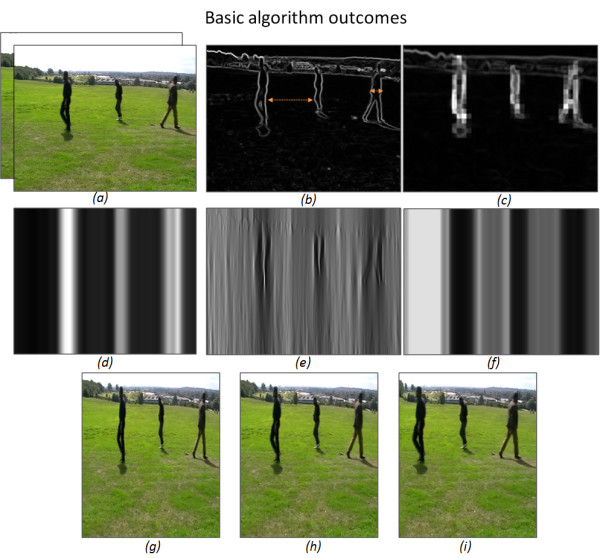
**Basic algorithm outcomes**. The output from each stage in our Seam Shrinking algorithm, when applied to two frames of video file. (a) is the two input images, (b), (c), (d) the spatial, spatio-temporal and modified importance matrix, respectively, (e), (f) the shrinkage map when using the unmodified and modified importance matrix, respectively, (g), (h), and (i) the retargeted image when using just interpolation, unmodified and modified shrinkage map, respectively.

Figure 2(b) and 2(c), show the generated spatial and spatio-temporal *importance *maps on a two-frame moving image. The arrows defining the space within the object and on the background represent areas of similar importance. Using the standard *shrinkability *approach described above would mean that both regions would be equally compressed. To give higher importance values to the entire body of such foreground objects, we would have to segment the entire image to determine which regions correspond to important bodies. This would require object recognition algorithms which are difficult and complex, requiring high levels of computational effort. Instead, we can adapt the seam carving approach described by Avidan and Shamir [15].

In the *seam carving *method, a cumulative energy map is generated based on the spatial *importance *map similar to that described in equation 3. We modify this to include temporal saliency as given in equation 5. We then search for seams with lowest energy and then give them very low importance values.

In an *n *× *m *image I, a vertical seam is defined to be

(6) $$\begin{array}{l} {\text{S}}^{\text{X}}={\left\{{\text{S}}_{\text{i}}^{\text{x}}\right\}}_{\text{i}=1}^{n}={\left\{\left(\text{x}\left(\text{i}\right),\;\text{i}\right)\right\}}_{\text{i}=1}^{n},\\ s.t.\forall \text{i},\;|\text{x}(\text{i})-\text{x}(\text{i}-1)|\;\;\leq 1 \end{array}$$

Where x(i) represents the column number for a given row, as the seam path should has only one pixel in each row of the image. This condition |x(i) − x(i − 1)| ≤ 1 is to make sure that the pixels along the seam path are connected.

Similarly, a horizontal seam is defined as follows:

(7) $$\begin{array}{l} S^{\text{Y}}={\left\{S_{j}^{y}\right\}}_{j=1}^{m}={\left\{\left(j,\text{y}\left(j\right)\right)\right\}}_{j=1}^{m},\\ s.t.\forall j,\;|y(j)-y(j-1)|\;\;\leq 1 \end{array}$$

As with the Avidan and Shamir [15] approach, we define the energy of a seam as the sum of all pixels' energy in this seam path. We then use dynamic programming to look for the seam with minimal energy. Taking the vertical seam as an example, we establish the cumulative energy matrix *M*:

(8) $$\begin{array}{l} \;M(i,j)=W_{ST}(i,j)\\ +min\{M(i-1,j-1),\;M(i,j-1),\\ M(i+1,j-1)\} \end{array}$$

Then starting from the last row in the *M *matrix, we search for the minimal cumulative pixel. After that, we work backwards from this pixel to obtain an optimal vertical seam by finding the minimum of the three neighboring pixels of this pixel in the previous row and then save this pixel to the seam path. At this point, Avidan and Shamir [15] would adjust the image width by removing this optimal vertical seam. These steps are repeated until the desired size is achieved.

At this point our method deviates from Avidan and Shamir algorithm in that we do not remove the seams; instead we save the locations of the pixels along the path of the all seams, and we generate another matrix *U *of size N×K, as K is the number of columns by which the input image should be shrunk. The *U *matrix contains the ***x ***and *y *positions of the K seams.

(9) $$U(S^{X}(i,j))=(i,\text{x}(i))$$

By knowing the locations of lowest energy pixels, we rescale all the pixels along the path of all the seams to very low importance values as following:

(10) $$W(U)=0.001*\frac{W(U)-\min(W(U))}{\max(W(U))-\min(W(U))}$$

**Figure 3:**
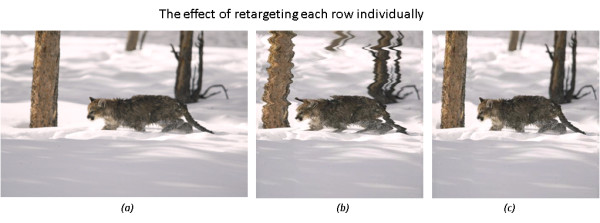
**The effect of discontinuity of rows**. (a) A 400 × 650 image. The image is retargeted to 400 × 400 by shrinking each row independently (b), causing a Zig-Zag, and when preserving row continuity (c).

The next step after adapting the *importance *matrix is to shrink the source image based on the importance of each pixel. However, if we simply shrink each row of the image independently, this would cause unnatural artefacts to the image content by creating a zigzag effect as shown in Figure 3(b). Thus, we have to ensure that *W*(*i*, *j*), is equal to *W*(*i*, *j *- 1), in case of horizontal retargeting, in order to preserve continuity between rows. To do that, we assign a fixed importance value for each column by applying a moving average window of size *L *and overlapping by *L*/2 on each column in the *importance *matrix [24], and then taking the maximum of these average values for each column as following.

(11) $$\begin{array}{c} {\bar{W}}_{r}(j)={\bar{W}}_{r-1}(j)+\frac{1}{L}[W_{r}-W_{r-L}],\\ r=1,...,\frac{2N}{L} \end{array}$$

Where ${\bar{W}}_{r}(j)$ is an array containing the moving average values of the importance values in column *j*, then the updated *importance *matrix is:

(12) $$W(:,j)=max_{r=1}^{2N/L}\{{\bar{W}}_{r}(j)\}$$

In the case of areas of very low importance beside very high importance ones, such as moving objects, then the final retargeted image will have some discontinuity artefacts. To remove any discontinuity in the *importance *matrix, we convolve it with a Gaussian smoothing function [24] which blurs the edges of the significant boundaries in the *importance *matrix. The final updated matrix is:

(13) $$W(i,j)=\frac{1}{2\pi \sigma^{2}}e^{-(i^{2}+j^{2})/2\sigma^{2}}*W(i,j)$$

Figure 2(b), (c) and 2(d), show the spatial, spatio-temporal and our modified *importance *matrix, respectively.

#### C) The shrinkability matrix

The *importance *matrix defines which pixels in the source image are important and should be preserved in the retargeted image. In contrast, the *shrinkability *matrix defines the relative extent to which pixels in the source image should be shrunk to achieve the target image. Because the values of all the pixels in each column of the *importance *matrix are the same, then we compute the *shrinkability *values of one row only and then replicate for the remaining rows. This considerably reduces the processing time. The *shrinkability *of a pixel means the reduction in that pixel's width when reducing the source image width by K pixels. If we consider K equal to 1, which means reducing the size of the source image by only one pixel. Then the shrinking value of each pixel *S*(*j*)can be calculated from this equation:

(14) $$S\text{(}j)=\frac{1}{W(j)*\sum_{j=1}^{M}1/W(j)}$$

The summation of *S*(*j*) over *j *columns equals 1 if K is 1. For higher values of K, i.e.number or columns to be removed/shrunk, the summation of *S*(*j*) is equal to K. This equation is repeated for the rows.

### D) Scaling the Shrinkability matrix

**Figure 4:**
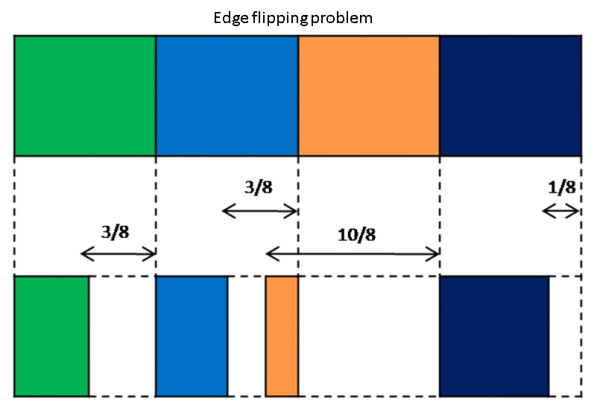
**Illustration of the edge flipping problem**. When the shrinkability value exceeds 1, the edge of the shrinkage pixel will move leftward past its left-hand edge. The number above each arrow indicates the value by which the pixel should be compressed.

To shrink the width of the source image by K columns, we can simply multiply the *shrinkability *matrix by K. However, this will result in some of the pixels in the scaled matrix exceeding 1. This means that these pixels would be moved to the position of the left neighbour pixels. Such pixel movements can cause, unnatural artefacts in the retargeted image, and is known as the edge flipping problem [18] which is described in Figure 4.

To solve this problem, we take the following steps:

(15) $$S^{'}(J)=K*S(J)$$

(16) $$S^{'}(J)=\min(S^{'}(J),0.9)$$

(17) $$S^{'}(J)=\left(K/\sum S^{'}(J)\right)*(S^{'}(J))$$

We repeat equations (16) and (17) until the summation of S'(J) equals to K. To avoid the total removal of any pixels, the threshold value is set to 0.9 and not 1. This method is much faster and more accurate than the one described by Zhang et al. [18], which applied a binary search method to find the best K_o _value that is close to the required K.

Figure 2(e) and 2(f), show the scaled shrinkage map when using the unmodified and modified *importance *matrix, respectively.

### E) Remapping algorithm

The scaled cumulative *shrinkability *is used to resample the source image to the retarget image by using an algorithm suggested by Karl M. Fant [25]. The algorithm is a 1 D method used in separable transformations defined in terms of forward mapping functions. It maps a limited line of discrete input pixel intensity values into a limited line of discrete output pixel intensity value. A full description can be found in Fant's paper, but we will summarise it briefly here.

The mapping is determined by a variable sizing factor of the output line in relation to the input line and by a position factor in relation to the output line. It can be characterized by the following formulation;

Each consecutive output pixel=S(consecutive input pixels).

Where S is a variable scaling factor, a size factor of the output data in relation to the input data which changes from pixel to pixel in the same line and 1/S is the inverse of the size factor which indicates how much of an input pixel contributes to each successive output pixel.

Figure 2(g), (h) and 2(i), show the retargeted image when using the ordinary interpolation method and when using the unmodified and modified *importance *matrix, respectively.

### F) Dynamic scene retargeting

To extend our algorithm into dynamic scene retargeting, we take into consideration some constraints from the previous frame. As previously mentioned, calculating the *importance *map for each frame individually generates jittering artefacts in the retargeted video sequence. We therefore start by considering spatio-temporal *importance *maps rather than considering the spatial importance alone (equation 4). If we relied purely on the spatio-temporal *importance *map, seams could cross to the other side of a significant feature, causing significant jitter. To counteract this, movement of seams from one scene to the next need to be constrained.

We therefore calculate the seams of the first frame or first couple of frames, and then the actual locations of these seems are stored into an arbitrary matrix *T *to be used in calculating the seams for the next frame. These seams' locations are adapted, for the forthcoming frames, if there are dynamics in the scene. If the objects in the frame are static then the locations of the seams will be the same, but if these objects move then the seam locations within the same areas through which the objects move around will also move to avoid crossing the moving objects.

For each seam, we first measure the difference between the energy of the stored seam path of the *importance *matrix in the previous frame and the energy of the same path of the *importance *matrix in the current frame. If the difference exceeds a threshold value *Z*, we search for another seam in the whole frame that has lower energy, and if the difference does not exceed *Z *then we search for a seam that might have energy less than the energy of the previous seam within a window of size Nx3 in the cumulative energy map *M*(*i*, *j*). If the energy of the new seam is greater than the previous one then we keep the previous seam intact. A summary of this process is shown below:

$$\begin{array}{l} For\;each\;frame\\ \;\;If\;\{\Sigma W_{current}(T(i))-\Sigma W_{previous}(T(i))\}>Z\;for\;i=1:K\\ \;\;Then\;search\;for\;new\;seam\;in\;M_{current}\\ \;\;Else\\ \;\;Search\;for\;new\;seam\;in\;M_{current}(T(i)-1,T(i),T(i)+1)\\ \;\;If\;\Sigma W_{current}(T_{new}(i))>\Sigma W_{current}(T(i))\\ \;\;\;\;\;\;Then\;use\;old\;seam\;T(i) \end{array}$$

By iteration, we have found that the best threshold value Z is 20% greater than the energy sum of the previous seam. Lower values of Z result in more frequent re-evaluations of the seams. This is more sensitive but results in jitter effects. Higher values of Z, increases stability with respect to jitter, but could result in distortion of the geometry of significant features.

### G) Downsampling to improve computational efficiency

There is one additional advantage of our algorithm over the *seam carving *method. In the *seam carving *approach, the process of generating the energy map and searching for the seams should be applied on the full size of the image to remove the seams repetitively. However, in our approach we generate seams to create an *importance *map rather than removing them from the scene. Hence, we can resize the source image into smaller one and generate the *importance *matrix from this downsampled image. After that, we resize the *importance *matrix into the original size of the source image. For example, if we down sized the source image by half, the overall processing time will be reduced by more than 60%, as most of the processing time is spent in generating and updating the *importance *matrix. This approach works well for high contrast scenes, though less well for more complex lower contrast scenes.

## Evaluation of Performance

The evaluation process is divided into two sections; synthetic evaluation based on testing the performance of our algorithm compared to other retargeting methods, and evaluations based on testing our algorithm on real subjects.

**Figure 5:**
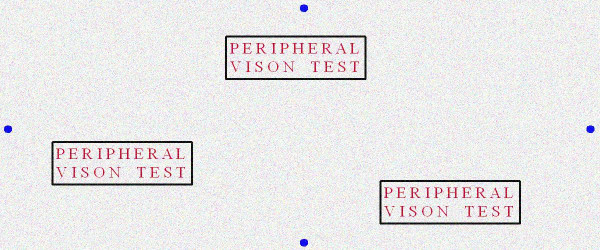
**Snapshot of our synthetic test video**. Showing three text boxes and blue alignment marker in the four directions; top, down, right and left.

From a comprehensive review of the literature it is clear that there is a lack of objective assessment tools to test efficacy of image rescaling algorithms. We have therefore generated a synthesized video (600 × 256) containing a three moving text boxes of the sentence "Peripheral Vision Test". The three boxes moved throughought the video space, from left to right and top to bottom. Gaussian Noise was added to investigate the effect of image texture complexity. Figure 5, shows a snapshot of the synthesized video.

If not done so already with automatic gain control, the video's frames luminance intensities are normalized between 0 and 255. We then applied our video retargeting algorithm for different retargeting sizes on this video file, from 20% to 62% of the original size, and did the same for the *seam carving *and *shrinkability *methods. The efficiency of the three algorithms was compared with respect to the ordinary video resizing by interpolation. For comparison, and to avoid subjective human interpretation, we measured three objective parameters:

### 1) Recognisability with respect to compression ratio

To measure this parameter we used optical character recognition (OCR) to count the number of correctly recognized words along the whole video frames. We developed our OCR interpreter based on the character template matching approach [26].

### 2) Compressibility

We measured the ratio between the average sizes of the three text boxes; when using the three algorithms, and when using the interpolation method.

### 3) Alignment

In the original video file we inserted four small blue circles (top, bottom, left and right). We measured the average vertical and horizontal displacement of these circles along the whole video frames to determine alignment changes after processing.

In addition, our algorithm was objectively and subjectively evaluated on real subjects. In the objective evaluation, our algorithm was tested on 20 volunteers using a simulated tunnel vision. Two pair of goggles, covering the whole eye area, was artificially painted, apart from an aperture, to simulate 10° and 20° tunnel vision effects. Objective testing was divided into 4 sections.

The first section was to assess the affect of our algorithm on speed of recognition. 26 images were displayed to the participants (13 with original size and 13 retargeted to 53% using our method). Images were projected to the participants using the NEC LT280 projector with resolution of 1024 × 768 and maximum projection brightness (At a distance of 2 m from the projection wall) of 2500 Lumens, in a darkened room. The projected area for the original image was (225 × 180 cm) which equivalent to 70° field of view. The 13 images included 3 synthetic images with colored shapes of squares, triangles and circles. The remaining 10 real-world images included football players with colored shirts on a grass background. Participants were asked to count the number of certain colored shapes (synthetic images) or shirts (real-world images) and allowed to move their head freely. The time taken to count these objects was measured for both of the original and retargeted images. This experiment was done for two simulated tunnel vision degrees; 11° and 22° field of view (FOV), respectively. Of the 20 participants, 6 were used as control subjects to do the same task without wearing the goggles. Those 6 subjects did both the whole test and the control part. Samples of the projected images are presented in the Appendix.

The second section of the test measured the efficiency of our method compared to the ordinary (linear) rescaling method. Three different images were compressed to 10%, 20% 40% and 50% of the original size using standard rescaling and our seam assisted shrinkability algorithm. The images were projected to the participants starting from the smallest size (10%) to the largest, and the ordinary rescaled images were displayed before our retargeted images. Participants were able to move their head freely and asked to count the number of colored shapes and the number of people in the images. The percentages of recognized objects were measured with respect to the total number of objects (total of 21 objects in the 3 images) in each scale for the ordinary resized images and our retargeted images.

The third section determined to what extent the compression algorithm could increase the effective field of view. The participants were asked to watch 3 video files (1 synthetic and 2 real-world) which included specific actions and objects. The participants were asked to count the number of times they perceived certain events in their peripheral field. The test was repeated for 40% retargeted versions using our algorithm. The percentage of detected objects was measured with respect to the total number of objects and actions (a total of 17 objects and actions occurred in the 3 video files) for both, the original and retargeted video. In two of the videos, the participants were asked to follow the moving object and in the third one they were free to move their heads. A Full description for the three video files used in this section is explained in the Appendix.

The last section measured the efficiency of our method over the ordinary rescaling method in recognizing certain actions in video. We compressed video files to 25%, 35% and 45%, respectively, of the original size using the ordinary rescaling and our method. The video was projected to the participants starting from the smallest size (30%) to the larger one, and the ordinary rescaled one is displayed before our retargeted one. The subjects were allowed to move heads freely. The percentages of recognized actions were measured with respect to the total number of actions (total of 4 actions) in each scale for the ordinary resized and our retargeted version. The description of the 4 actions is presented in more detail in the Appendix.

The subjective test of this part is divided into two sections. Firstly, the performance of our seam assisted shrinkability method was subjectively compared to the seam carving and shrinkability only methods on healthy subjects. We then separately performed a comparison of our seam assisted shrinkability algorithm with ordinary (linear) resizing. This second study is to explore the tradeoffs' between improved object sizes with inevitable levels of jitter. 6 video files were used in this evaluation, which were each retargeted with the three algorithms plus retargeting them with the ordinary resizing technique. The first video sequence consisted of four subjects passing a tennis ball to each other while they are standing and not moving. In the second video sequence, 5 subjects pass a basket ball while they are moving in a circular form. The third video consists of a subject moving (in the middle of the scene) towards the screen from a distance while three other subjects enter and exit from the frame. In the fourth video, a subject is playing with golf balls, so the motion in this video was considerably slow. The fifth video consists of a subject skating on the water. The final video consisted of eight subjects dancing in a small room. This was the busiest of the 6 videos.

Table 1 shows the dimensions of each video file and the rate of compression applied. For the first part of the test, the retargeted videos were shown to 14 normal subjects who were asked to select the most subjectively preferable. For the second part, subjects were asked to select the most preferable one between the most preferable from the first part and the ordinary resized videos. Subjects were asked to consider their judgement based on the degree of object distortion, and jitter between frames.

**Table 1:** Sizes of the original and retargeted videos used in the preference experiment

Video number	Original size	Retargeted size	% Compression
1	240 × 320	240 × 180	43.75
2	240 × 320	240 × 200	37.5
3	240 × 320	240 × 200	37.5
4	400 × 700	400 × 300	57.14
5	210 × 320	210 × 160	50
6	200 × 420	200 × 200	52.38

All the P values mentioned in the next sections are calculated based on the one tail t-Test.

## Results

We present the results in two sections; the synthetic performance of our algorithm compared existing methods, and results obtained from human testing with simulated tunnel vision.

### A) Results from synthetic testing

**Figure 6:**
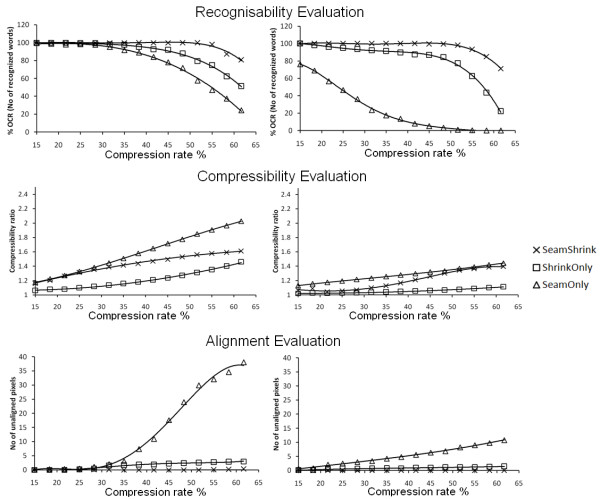
**A comparison between the performance of seam carving, shrinkability, and our method by measuring the three parameters**: The OCR (top row), the compressibility (middle row) relative to the ordinary interpolation, and the Zig-Zag effect. The left column shows the performance when σ of the added Gaussian noise (simulation to the contrast between the foreground and background) is 0.025, and the right column when σ is 0.45.

**Figure 7:**
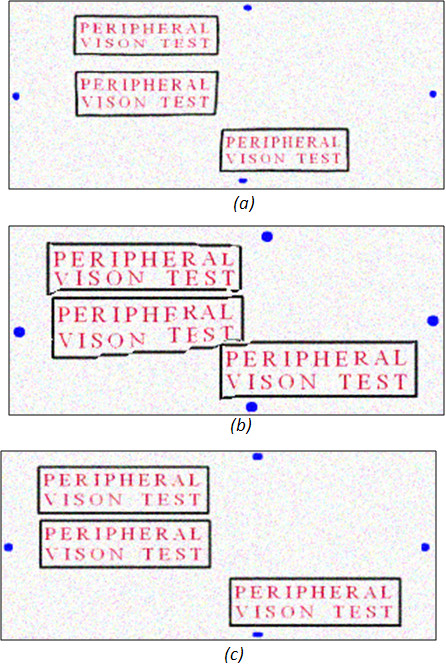
**Comparison between the retargeting of the frame by 50% of its original size when using**. The shrinkability algorithm (a), the seam carving algorithm (b), and our seam shrinking algorithm (c).

Figure 6, shows the performance of our algorithm compared to the *seam carving *and the *shrinkability *methods in respect to the three measuring parameters mentioned previously. The left column shows the performance for low added noise (σ = 0.025), and the right for high added noise (σ = 0.45), respectively. We can see that our algorithm has the highest OCR recognition values over the range of compression rates. This is because it combines the advantages of preserving the size of the text boxes, which are the foreground objects in this case. Additionally, it maintains the connectivity of these objects without distortion. From Figure 6 middle row, we can see that *seam carving *has the highest compressibility, but this efficiency in compression is to the cost of increased distortion, and reduced connectivity and stability of the objects as shown in Figure 7. This is because seams tend to go through higher energy pixels, if higher compression is required. Figure 6 last row, compares the number of unaligned pixels for the three methods. The *seam carving *alone causes more zig-zag and misalignment effects than our algorithm and the *shrinkability *method while increasing the compression rate.

**Figure 8:**
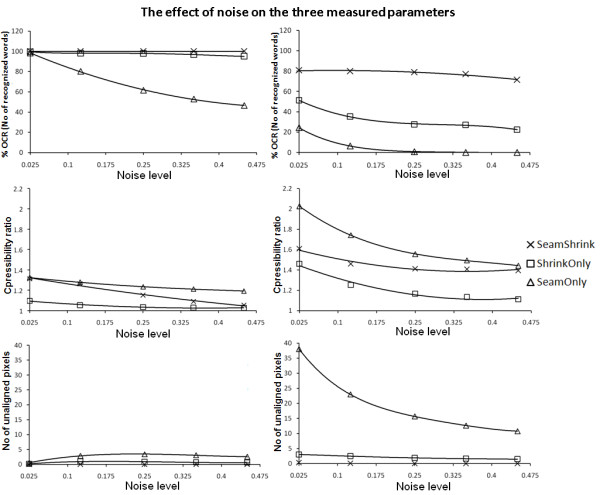
**The effect of increasing σ of the added Gaussian noise (to simulate the variation on contrast between the foreground and background)**. When 25% compression rate from the original size is required (left column) and 62% compression rate (right). The first row is for the OCR measurement, the second row is for the compressibility measurement, and the last row is for the Zig-Zag or unalignment measurement.

Figure 6 and Figure 8, show the effect of increasing the σ of the added Gaussian noise on the efficiency of our method compared to the *seam carving *and the *shrinkability *methods in respect to the OCR, compressibility and unalignment measurements, respectively. From the top row of Figure 6 and Figure 8, we see that the OCR efficiency drops sharply with increasing the noise levels in the case of *seam carving *method. This is because adding more noise blurs the foreground/background contrast and hence the energy map will tend to be smoothed with slightly equal weights. In the case of the *shrinkability *method, the drop in the OCR efficiency starts to increase sharply when a compression rate of more than 45% is used. Our method shows a slight drop in the OCR efficiency with compression, but not as much as the other two methods.

The middle row of Figure 8 shows the effect of increasing the noise level on the compressibility performance. When a small compression rate is required and the noise level is low (which means the background gradient is low), our seam assisted shrinkability method efficiency is high. This is because the seams will be accumulated in the low gradient areas, causing these areas to be shrunk more. Increasing the noise level (gradient of background becomes high) drops the compressibility performance in all algorithms. Our algorithm performs better than shrinkability only, but starts to approach this latter algorithm at high noise levels during lower compression.

Also from the last row of Figure 6 and Figure 8, we see that the number of unaligned pixels tends to decrease with increasing the noise level and when high compression rates are required. This is because increasing the noise level gives the pixels increasingly equal energy, resulting in some of the seams crossing into the text box. Hence, the seams will tend to go straight from down to top causing less misalignment effect.

**Figure 9:**
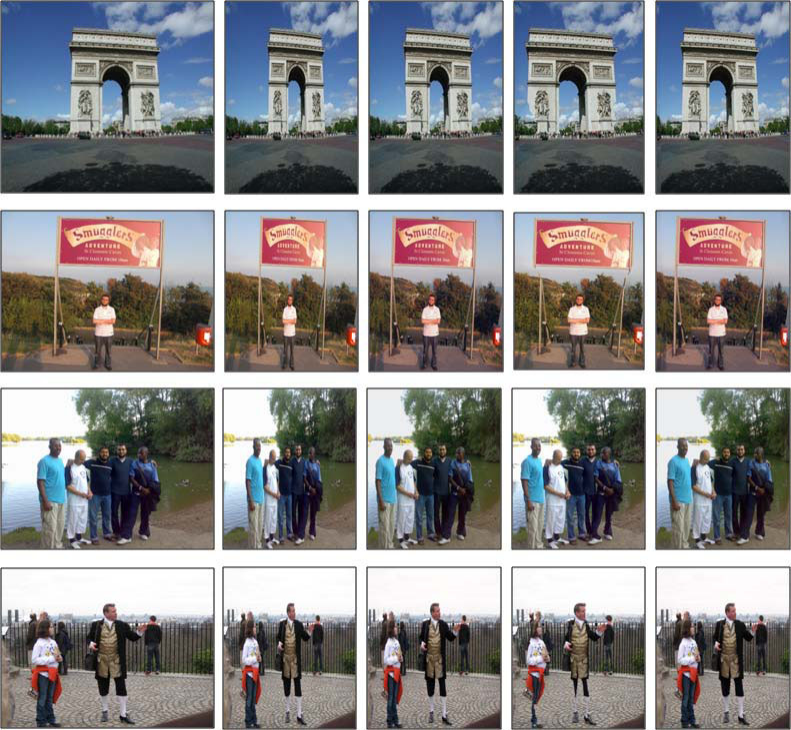
**A comparison between different image retargeting techniques and our method**. From left to right: original, interpolation, shrinkability, seam carving, and our seam assisted shrinkability method. All the images are retargeted to 62% of the original size.

Figure 9, shows the retargeting of different images when using the traditional image resize, *seam carving*, *shrinkability *and our seam assisted shrinkability method. Retargetting was performed on the horizontal direction only for these images to compare effects. *Seam carving *results in effective scene compression up to a certain target width. However, this is on the cost of removing parts of the foreground objects, causing severe distortions in these objects. Since seams have irregular shapes, the distortion is unpredictable. Alternatively, our proposed algorithm compensates these distortions by compressing the pixels instead of removing them.

### B) Results from human trials

**Figure 10:**
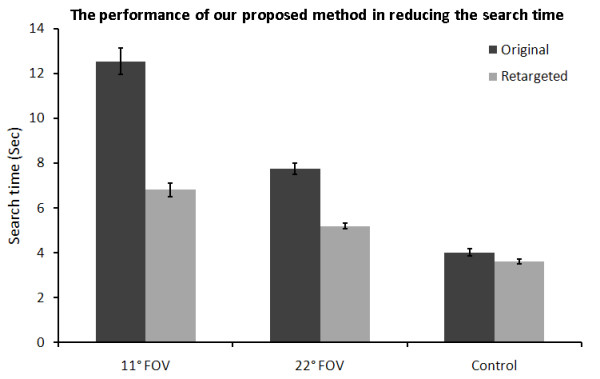
**The efficiency of the seam assisted shrinkability method on search time**. We simulated the effect of tunnel vision by asking users to wear blacked out goggles with apertures corresponding to 11° and 22° FOV. Greater improvement on search time improves as the tunnel vision worsens. Mid-Late stage RP typically expresses tunnel vision in the 5° to 20° range. The error bars represent the standard error of the data.

Results from the real subjects are divided into objective and subjective results. Objective's results are divided into 4 sections, as described in the evaluation section. The first two sections are for the still images and the third and fourth sections are for the dynamic scenes. The time taken for counting the number of objects within the 26 images is shown in Figure 10. We can see that there is a significant reduction (P < 0.001) in the time taken to count the objects of the retargeted images by approximately 50% in the case of 11° FOV goggle. Also there is a significant reduction (P < 0.001) in the time by 35% when using the 22° FOV. Even then, there was a relatively significant (P < 0.035) difference between the times taken in the original sizes and the retargeted ones in the control subjects, which due to the peripheral vision effect. As the participants were still needs to move their eyes around the images while counting, while in the retargeted images they do not need to move their eyes.

**Figure 11:**
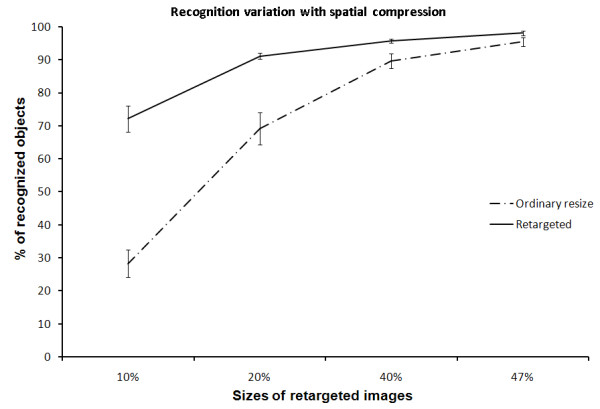
**Recognition variation with spatial compression**. The efficiency of retargeting the images using our seam assisted shrinkability method compared to the ordinary resizing method in recognizing objects, when scenes were compressed into 10%, 20%, 40% and 50%, respectively, of their original sizes. The error bars represent the standard error of the data.

Figure 11, shows the efficiency of our compression method over the ordinary (linear) rescaling method, when compressing the images to 10%, 20%, 40% and 50% of their original size. We can see that, using our compression technique, more than 72% of the objects in the images are still recognizable when compressing the image into 10% of the original size, compared to only 28% when linearly compressed. This gives more than 150% enhancement in recognition over the ordinary resizing. The efficiency gap became very small at 50% compression.

**Figure 12:**
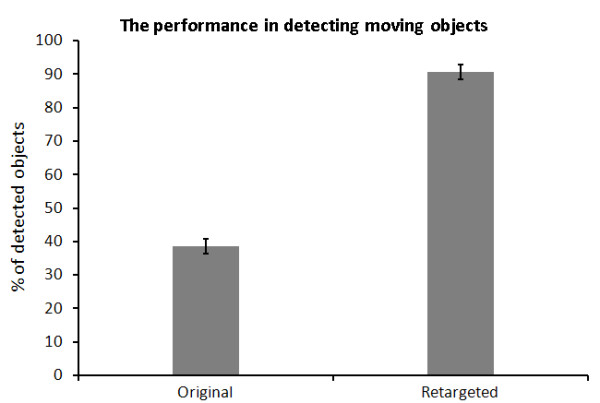
**The efficiency of detecting objects**. The efficiency of detecting objects when retargeting dynamic scene into 40% of the original size using our seam assisted shrinkability method compared to the original size. The error bars represent the standard error of the data.

In the case of dynamic motion, Figure 12 shows the efficiency of detecting and counting moving objects when the scene retargeted to the field of view. As shown from the figure, there is a significant difference (P < 0.001) between the counted objects in the original videos and the retargeted videos. This gives an enhancement of object detection by more than 136% when retargeting the scene.

**Figure 13:**
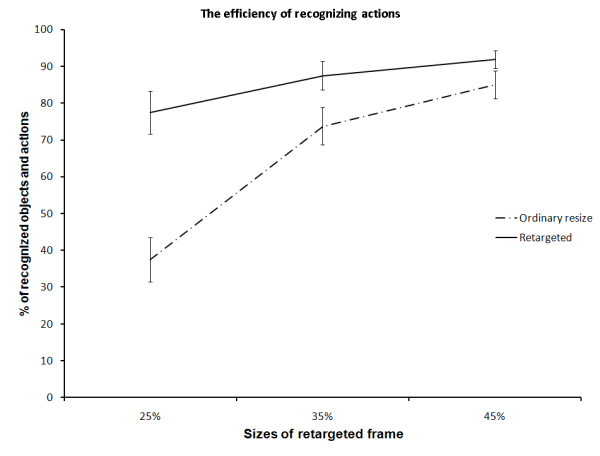
**The efficiency of recognizing actions**. The efficiency of recognizing actions when compressing the dynamic scene into 25%, 35, and 45% of the original size, using the ordinary resizing method and our method. The error bars represent the standard error of the data.

Similar to the observations from Figure 11, Figure 13 shows the efficiency of our compression method over the linear rescaling method, when compressing the dynamic scene into 25%, 35% and 45%, respectively, of their original size. More than 77% of the actions are still recognizable at 25% of the original video size when retargeted using our method, compared to only 37% when linearly resized. This gives more than 100% enhancement in recognition process over the ordinary resizing. However, the efficiency gap tends to decrease when reducing the compression rate.

**Figure 14:**
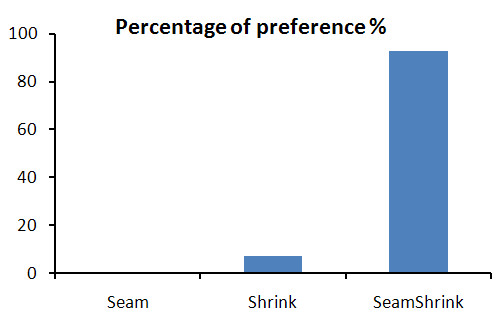
**Subjective preference test**. The preference test is between our method and the seam and shrikability methods. The test was done on 14 subjects showed to them 6 retargeted videos using the three methos.

**Figure 15:**
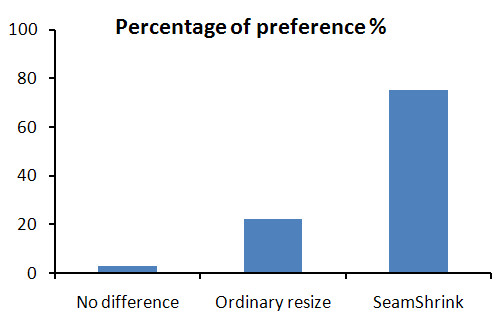
**Subjective preference test**. The preference test is between our method and the ordinary resized method.

Results obtained from the subjective preference test of our method, the *seam carving *and *shrinkability *only methods are shown in Figure 14. The results show the average preference over the 14 subjects for the 6 dynamic movies. Overall, the retargeted videos by the seam assisted shrinkability method were preferred by 93% compared to the shrink and seam only methods, 7% and 0% respectively. This result shows good evidence on the efficiency of our proposed method over the previous two methods. In the second part of the subjective test, our seam assisted shrinkability method was compared with the ordinary resized videos. Figure 15 shows the result of this evaluation. Overall, our method was preferred by 75% compared to 22.22 of the ordinary resized videos. For the remaining 2.78%, the subjects couldn't see much difference between the two retargeting methods. These results show that the low levels of jitter resulting from our retargeting method is acceptable given the added benefit.

## Discussion

The synthetic results indicate that our method is superior to both the standard resizing method and previous seam carving and shrinkibility retargeting methods. Simulated patient trials indicate that greatest benefit is achieved in those of remaining field of view less than 20°. The algorithm would therefore be also useful in retinal prosthetic devices [27] which will in initial implementations only return a limited visual field. The recent optogenetic approaches [27] look particularly promising and could potentially return higher resolutions. However, assuming the technology can approach a level of development of present head mounted displays for virtual reality, the fields of few will still be limited to typically 40°. Thus some form of compression would greatly assist such users. As the visual acuity may still be low, scene simplification and enhancement operations may additionally be needed [28].

**Figure 16:**
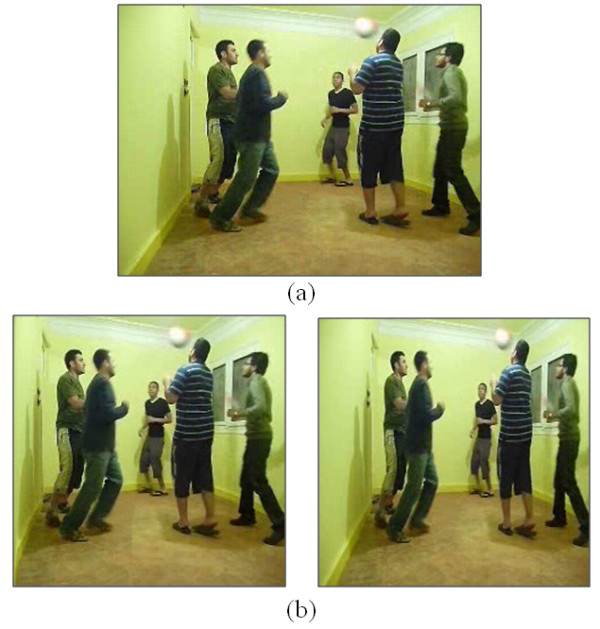
**Limitation of the algorithm for busy environment**. (a) Snapshot of a busy video for guys playing with basketball in a very small area. (b) Shows a 25% compression on it, when compressed linearly (right) and with our retargeting algorithm (left).

Importantly for application to augmented reality visual aid devices, this algorithm can dynamically compress video sequences without knowledge of future frames. While low levels of jitter are ever present, we believe that at moderate compression rates the jitter is sufficiently low to be acceptable to our target patient group. Although this has not yet been tested in patients, subjective tests in healthy subjects show indicate efficacy. The level of jitter increases when large compression is required in highly dynamic and busy environments, such as the one shown in Figure 16. This figure shows a snapshot from a video of guys playing with a basketball in a very small area. For such environment, a 25% compression from the original size can give acceptable results. However, in less crowded environments, up to 60% compression can be achieved with low levels of jitter. We would thus envisage patient's being able to control levels of compression rate according to the surrounding environment and personal preference.

Ultimately, this work has shown efficacy in scenes whereby the camera is static and objects in the scene move. In this situation creating an energy map for the motion energy is straight forward and obtained from the movement of objects across the frames. However, in the situation where the camera moves relative to the environment, the movements of all features would need to be subtracted from the background movement. This background movement would then have to be calculated from optic flow analysis on the images or from accelerometer motion sensors.

A further point to be considered is that this presented algorithm has been designed for monocular vision. In binocular cases, the situation is more complex as slight differences in left and right scenes may vary the compression such that objects to not appear to overlap in 3D space. We will thus look to further develop this technique in future for binocular use.

Key to implementability of any retargeting algorithm is its function on portable processing systems at full video frame rate. We used the Matlab platform and the C language to build our seam assisted shrinkability algorithm. The processing was implemented on a desktop computer; HP workstation XW4600 with a 2.6 GHz Intel Core Quad processor. We did not use any GPU assisted processes and thus all the operations are processed through the CPU/FPU. Table 2 shows the processing time of the major parts in the algorithm when retargeting one frame of size 352 × 288 by 43%. The table shows the processing time when downsampling the input frames into different scales to calculate the *importance *map.

**Table 2:** Downsampling effect on the processing time

Downsampling by	Processing time (sec)			
	Importance map	Shrinkability & Fant	Other functions	Total
1	0.8	0.02	0.03	0.85
2	0.17	0.02	0.03	0.22
4	0.05	0.02	0.03	0.1

We can see that the processing time of the *importance *map drops approximately by 4 when downsampling the input frames by half of the original size. However, the time taken in generating the *shrinkability *map and remapping by Fant's algorithm is fixed because these processes applied on the whole size of the input frame.

Currently, all of the algorithm's parts are running serially on the computer CPU. However, some parts of the algorithm can be optimized to run in parallel on a graphic processing unit (GPU) or even on a portable field programmable gated array (FPGA) device [29]. For example, most of the *importance *map parts are based on the convolution kernels which can be implemented in parallel. Not only can the importance map be parallelized, but also the process of generating and scaling the *shrinkability *map. Such parallel processing can be performed in portable GPU or FPGA devices. Because the Fant's algorithm remaps each row individually on the retarget frame, it is also possible to implement this in parallel architecture. In future work we hope to implement this algorithm on a portable parallel processing platform so as to perform real time RP patient trials. As is generally accepted in the graphics processing community, parallel processing using GPU architectures can speed up the processing time in the range of 10-100× depending on the level of parallelism of the program. Although 25frames per second is sufficient for video rate, 50 frames per second is widely accepted as the minimum to reduce strain through motion blur. We believe this target can be achieved in portable GPU architectures.

## Conclusions

In this article we have described a novel content aware scene retargeting technique developed for patients suffering from retinitis pigmentosa. Our approach tackles the shortcomings of the two current techniques for image retargeting (*seam carving *and *image shrinkability)*. In particular we have improved upon issues such as discontinuity artefacts and jitter in real time video sequences.

We combined the advantages of both techniques, by designing a novel *importance *map which gives the pixels along the seams paths lower importance values. Then we used the modified *importance *map to build a *shrinkability *matrix to shrink the pixels according to their shrinkage values.

Results show the robustness of our approach compared to the *seam carving *and image *shrinkability *techniques in preserving the scene contents intact and in the compressibility performance.

## Consent

Written consent was obtained from the participants for publication of this paper and accompanying images. A copy of the written consent is available for review by the Editor-in-Chief of this journal.

## Competing interests

The authors declare that they have no competing interests.

## Authors' contributions

WA developed the algorithms, TT developed the image shrinkability component. WA and PD performed the data analysis and manuscript writing. All authors read and approved the final manuscript

## Appendix: Description of the trials

**Figure 17:**
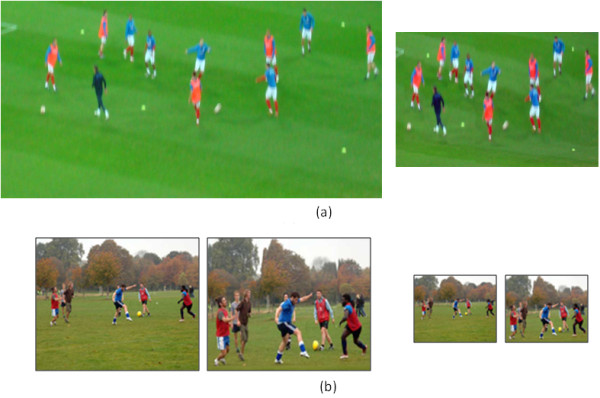
**Samples from the trial**. Samples of the images used in the first section of the test (a). And in the second section (b), where the image is compressed to 10% (right) and 20% (left) of the original size, respectively. The left image in each pair of images in (b) is the one compressed linearly and the right one is the retargeted using our method.

To evaluate the efficiency of our method in reducing the searching time for specific objects, we know that the selected images in the trials should have the same number of objects before and after retargeting. It is not easy to select very similar images that have the same complexity and number of objects to compare between them. For that we selected the images of the players in the playground to be used in this trial, which eliminate the effect of memory. Figure 17(a), shows a sample of the used images in the first section before and after retargeting.

Figure 17(b), shows sample from the second part of the test. The lowest size of the image is displayed first to the participant to count the number of objects in the image (objects here is the total number of people), as shown in the right pair of images. Then the larger size is displayed to count the number of objects, as shown in the left pair. The image to the left in each pair is the one resized linearly and the one to the right is retargeted using our method, respectively.

**Figure 18:**
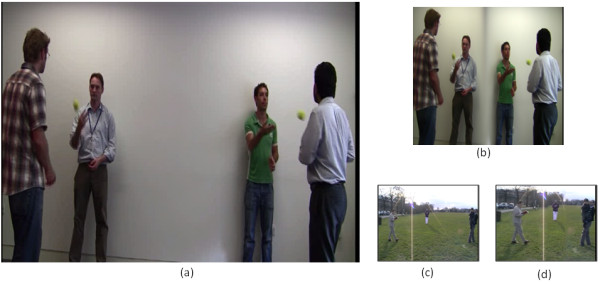
**Snapshots from the dynamic scenes**. Snapshots from the second video file in the third section of the test, before (a) and after (b) retargeting, respectively. Snapshots from the video used in the forth section of the test, where the video is compressed into 25% of its original size using linear resizing (c) and our retargeting method (d).

In the third section of the test, the first video shown to the participant includes a blue arrow moving from the left side to the right hand side. Participants are asked to focus on this moving arrow and count the number of blue squares appearing in their FOV, which are 3 blue squares in total. The second video file includes 2 pairs of persons passing two small tennis balls to each other. The participants are asked to count the number of passes of the tennis balls, which are 11 passes. In this video, the participants were free to scan the whole video while counting. In the third file, the participants are asked to focus on a walking person and to count the number of people appearing in their FOV, which are 3 in total. A 40% of the original size retargeted versions of these videos were shown to the participants to count the same objects. Figure 18(a, b), shows a snapshot of the second video file (people playing with a tennis ball) before and after retargeting, respectively.

The last section in the test includes only one video which compressed into 25%, 35, and 45% of its original size using linear rescaling and our retargeting method. The movie includes 4 persons moving around while doing specific actions (reading, drinking, eating a banana, and holding a child). The participants are asked to recognize the actions of these persons in each scale. Figure 18(c, d), shows a snapshot from the 25% version of the video, when linearly resized and when using our retargeting method, respectively.

## Note

NB: All the images and videos included in this manuscript have been taken by the author.

## Acknowledgements

We would like to acknowledge and thank The University of London Central Research Fund (AR/CRF/B), the Royal Society Research fund, the National Institute for Health research (BRC) fund and the EPSRC (F029241) for supporting this research. Also Mr. Walid Al-Atabany would like to thank the Egyptian government, who are sponsoring him for his PhD. We would also like to thank Dr. Muhammad Ali Memon and Dr. Susan Downes for our collaborations on past, present and future patient trials in this augmented vision area.
